# RBM24 Mediates Lymph Node Metastasis and Epithelial-Mesenchymal Transition in Human Hypopharyngeal Squamous Cell Carcinoma by Regulating Twist1

**DOI:** 10.1155/2022/1205353

**Published:** 2022-09-29

**Authors:** Yuhong Liu, Min Pan, Tao Lu, Yanshi Li, Dan Yu, Zhihai Wang, Guohua Hu

**Affiliations:** Department of Otorhinolaryngology, First Affiliated Hospital of Chongqing Medical University, Chongqing 400016, China

## Abstract

**Objective:**

Despite the target RNA regulatory action of RBM24 (RNA Binding Motif 24), a protein implicated in multiple carcinomas, its role in HSCC remains unclear. Our study probed to understand the effect of RBM24 on HSCC.

**Materials and Methods:**

A combination of qRT-PCR, IHC, and western blot was employed to assess the HSCC tissue level of RBM24. The colony formation and CCK-8 assays were performed to estimate cellular proliferative potential, whereas the transwell assay was conducted to examine invasive and metastatic potential. The FaDu cell motility was assessed via the scratch-wound assay and EMT (epithelial-mesenchymal transition) by adopting qRT-PCR in conjunction with western blot and IF (immunofluorescence). The in-vivo effect of RBM24 on HSCC was investigated through modeling metastasis to the popliteal LNs (lymph nodes).

**Results:**

Among HSCC patients showing metastasis to LNs, prominent RBM24 downregulation was noted, with an intrinsic association between low RBM24 level and poor outcome. Knocking down RBM24 promoted cell multiplication, migration, and infiltration, while overexpression led to the opposite effects and inhibited the EMT. RBM24's suppressive action against the FaDu cell mobility and invasion was reversed by Twist1 overexpression. RBM24's suppressive actions against the tumor evolution and LN metastasis in HSCC in-vivo were also validated.

**Conclusion:**

As a carcinoma inhibitor gene, RBM24 regulates Twist1 to achieve LN metastasis and EMT suppression in HSCC.

## 1. Introduction

As a highly invasive malignancy representing almost 0.4% of carcinomas [1], HSCC (hypopharyngeal squamous cell carcinoma), despite being rare, is prognostically inferior to any cancers of the head and neck, with a relative five-year survival between 25% and 45% [2–5]. According to Ho et al., a chief independent factor linked tightly to the mortality risk among patients with hypopharyngeal carcinomas was the metastatic LN (lymph node) count [6]. Although the diagnostic measures and therapeutic regimes have tremendously improved, the condition in HSCC patients usually progressed to an advanced stage upon initial diagnosis, showing cervical LN or distant metastasis, and the prevalence of LN metastasis was 65-80% [7, 8]. Thus, understanding the malignant progression mechanisms in HSCC with LN metastasis at the molecular level is necessary for early screening biomarkers to enhance diagnostic precision. Implicated in regulating the target RNA substrates nearly over the whole life cycle, RBPs (RNA-binding proteins) may exert a kernel function in gene expression networks [9]. An RBP census in humans demonstrated that 7.5% of genes encode proteins in gene expression modulation [10]. In addition, RBPs are considered carcinogenesis participants [11]. Thus, transcript modulation is achieved by classic RBPs through the formation of RNP (ribonucleoprotein) complexes with corresponding RNA targets through the RBDs (RNA-binding domains), including the hnRNP KH (K homology) domains, the RRMs (RNA recognition motifs), and the S1 domains [12]. An RBP, RBM24 (RNA Binding Motif 24), encompasses a highly conserved RRM consisting of 2 submotifs (RNP1 and RNP2) [13], which could promote the cardiomyocytic differentiation from embryonic stem cells through the alternative splicing modulation [14] and improve the myogenic differentiation through transcript stability regulation of myogenin mRNA by binding to its 3′-UTR (3′-untranslated region) [15] in mice. Although the role of RBM24 as a carcinoma inhibitor gene has been demonstrated in NPC (nasopharyngeal carcinoma) [16], CRC (colorectal cancer) [17], and hepatic carcinoma [18], its precise function in HSCC is elusive.

Owing to EMT (epithelial-mesenchymal transition), an important event linked tightly to carcinoma progression capable of mediating metastasis, the tumor cells can invade the paracarcinoma tissues and achieve metastasis [19]. Twist1, a member of the basic helix-loop-helix protein family, is a primary regulator of EMT. Upregulation of Twist1 facilitates the malignant progression of HSCC and is related to poor prognosis [20, 21]. In contrast, Twist1 overexpression makes the FaDu cells less chemically sensitive to Taxol [22], despite its unclear function in the LN metastasis in HSCC.

Our work is the first to discover RBM24 downregulation among HSCC patients showing LN metastasis. We aimed to prove the capability of RBM24 to suppress the multiplication and metastasis of HSCC cells both in-vivo and in-vitro. Besides, it could be demonstrated that Twist1 overexpression, RBM24's downstream target, rescues RBM24's suppressive actions against the FaDu cell invasion, migration, and EMT. These outcomes could reveal that RBM24 probably targets Twist1 to achieve metastatic LN inhibition.

## 2. Materials and Methods

### 2.1. Patients and Tissue Samples

In this study, fresh HSCC tissues and the corresponding adjacent normal tissues were derived from patients suffering from surgery at the First Affiliated Hospital of Chongqing Medical University and were maintained at −80°C to perform protein and RNA extraction. The surgical specimens for IHC were collected from patients who underwent surgery at the First Affiliated Hospital of Chongqing Medical University from 2015 to 2019. The criteria for inclusion were: (i) diagnosed with HSCC pathologically. (ii) Laryngectomy performed only once. (iii) Completion of Follow-up data and electronic nasopharyngoscopy, CT, or MRI examination was performed every six months after the initial operation. Besides, the exclusion criteria were: (i) preoperative radiotherapy, chemotherapy, or targeted therapy had been performed. (ii) The diagnosis was another type of tumor. Two experienced pathologists performed pathological diagnosis and tumor grading. In the end, 57 patients diagnosed with HSCC were included in the current work.

### 2.2. Immunohistochemistry (IHC) and Scoring

Paraffin-embedded tissue sections (4 *μ*m) were deparaffinized in xylene and rehydrated in a graded ethanol series. Sections were heated in citrate buffer for 15 min at 100°C for antigen retrieval. Next, slides were rinsed in phosphate-buffered saline (PBS) and treated with peroxidase block at 37°C for 10 min. After blocking with 10% normal goat serum at 37°C for 15 min to minimize nonspecific staining, the slides were incubated with an anti-RBM24 primary antibody (1 : 200; ab94567, Abcam, Cambridge, UK) overnight at 4°C. The next day, the slides were incubated with a horseradish peroxidase (HRP)-conjugated polyclonal goat secondary antibody for 15 min and incubated with diaminobenzidine (DAB) reagents (ZSGB-BIO, Beijing, China) at room temperature for 1 min for detection of antigen-antibody interaction signals. Finally, the slides were counterstained with hematoxylin for 20 sec. Slides incubated with PBS instead of the primary antibody were used as a negative control. All slides were visualized by two experienced pathologists. Determinants of IHC score included the ratio of tumor cells positive for RBM24 and the staining intensity. The first determinant was scored 0 for 0-5%, 1 for 5-25%, 2 for 25-50%, 3 for 50-75%, or 4 for 75-100%. The second determinant was scored 0 if negatively stained, 1 for weakly stained, 2 for moderately stained, or 3 for strongly stained. The product of the preceding two scores constituted the final IHC score. The RBM24 expression classification was accomplished by the ROC curve-based assessment of IHC scores, with a threshold of 4. The expression was considered low if the score < 4, whereas high if ≥ 4.

### 2.3. Cell Line and Culture

Human HSCC FaDu cells were procured from the Chinese Academy of Sciences (Shanghai, China) and maintained at 37°C and 5% CO_2_ conditions in the MEM (minimal essential medium) (Boster, USA) with 10% FBS (PAN-Biotech, Adenbach, Germany) and 1% penicillin-streptomycin.

### 2.4. Lentiviral Transduction

The GFP (green fluorescent protein)-expressing lentiviral particles for RBM24 overexpression, namely OE-RBM24 and Control, were procured from GeneChem (Shanghai, China). After reaching 20-40% confluency, FaDu cells were plated onto a 6-well microplate, followed by infection using lentiviral particles at a 10 MOI (multiplicity of infection). The cells were screened after 48-72 h of infection using MEM with 2 *μ*g/mL puromycin (Beyotime, Shanghai, China). Cells were further harvested for assessment via qRT (quantitative real-time)-PCR in conjunction with western blot.

### 2.5. siRNA Transfection

siRNA oligonucleotides against human RBM24 (Si-RBM24, 5′-GAG CUG CAU ACG CAC AAU ATT-3′ and 5′-UAU UGU GCG UAU GCA GCU CTT-3′) and a scrambled siRNA (Si-NC sense, 5′-UUC UCC GAA CGU GUC ACG UTT-3′ and antisense, 5′-ACG UGA CAC GUU CGG AGA ATT-3′) were obtained from GenePharma (Shanghai, China) to knockdown RBM24. In addition, FaDu cells were seeded into a six-well plate, and Lipofectamine RNAiMAX (Invitrogen, CA, USA) was utilized to transfect these siRNAs according to the manufacturer's instructions. In addition, the knockdown efficiency was examined using qRT-PCR and western blotting.

### 2.6. Plasmid Transfection

A Lipofectamine 3000 system (Invitrogen, Carlsbad, USA) was employed for the transfection of the PCMV (control plasmid) and OE-Twist1 (Twist1 overexpressing plasmid) (Tsingke Biotechnology, Nanjing, China), into the FaDu cells.

### 2.7. Western Blotting (WB) Analysis and Antibodies

Total proteins were sampled from FaDu cells and tissues via a kit (KeyGen, Nanjing, China). A BCA Assay Kit (Beyotime, Shanghai, China) was utilized to examine the protein concentration. Hybrid proteins were isolated via SDS-PAGE (10%), placed onto the PVDF (polyvinylidene fluoride) membranes (Biosharp, Hefei, China), subjected to a 15-minute blockage using buffer (Beyotime, Shanghai, China), and incubated overnight using 1 : 1000 anti-RBM24 (ab94567), 1 : 5000 anti-Vimentin (ab92547; both Abcam, Cambridge, UK), 1 : 1000 anti-E-cadherin (#3195), 1 : 1000 anti-N-cadherin (#13116; both Cell Signaling Technology, MA, USA), 1 : 1000 anti-Twist1 (AF4009; Affinity, Jiangsu, China), and 1 : 3000 anti-GAPDH (AF1186; Beyotime, Shanghai, China) antibodies at 4°C. Subsequently, 1 h processing of the membranes was accomplished using 1 : 5000 anti-rabbit goat IgG (A0208; Beyotime, Shanghai, China) at ambient temperature. Finally, an XRS+ imaging system (ChemiDoc™; Bio-Rad, CA, USA) was utilized to examine the specific proteins.

### 2.8. qRT-PCR Analysis

Total RNA was extracted using a Total RNA Kit I (Omega, Norcross, USA). An RT Reagent Kit with gDNA Eraser (Perfect Real Time) (PrimeScript™; Takara, Dalian, China) was employed for complementary DNA synthesis and assessed using qRT-PCR using an SYBR PrimeScript™ RT-PCR Kit (Takara, Dalian, China). The 2^−ΔΔCt^ approach was applied for the mRNA level assessment.  Table 1 presents the primer details.

### 2.9. Cell Proliferation Assay

Cell proliferation was examined using Cell Counting Kit-8 (CCK-8; GlpBio, Montclair, CA, USA) reagent. FaDu cells were plated at 2,000/well onto a 96-well plate. Subsequently, CCK-8 (10 *μ*L) was added to each well, and the 96-well plate was incubated at 37°C containing 5% CO_2_ for 1 h. Subsequently, the absorbance was assessed at 450 nm, and data were collected continuously for 96 h.

### 2.10. Colony Formation Assay

FaDu cells were plated at 1,000/well onto a 6-well microplate and incubated for 2 weeks in 10% FBS-MEM. The cells were then immobilized using methanol, stained with crystal violet (0.5%), and colonies were quantified, comprising a minimum of 50 cells.

### 2.11. Transwell Migration and Invasion Assays

Transwell assays were performed for the invasive and migratory capacity assessment of FaDu cells. The chambers were filled with membranes (pore size: 8 *μ*m; Corning, NY, USA) coated by Matrigel (BD Biosciences, MA, USA) or noncoated membranes. The upper chambers were added with 5 × 10^4^ cell-involving serum-free MEM (200 *μ*L), whereas the lower chambers with 20% FBS-MEM (600 *μ*L). Further, the cells were immobilized in methanol for 48 h later, subjected to the crystal violet (0.5%) staining, and quantified microscopically.

### 2.12. Wound Healing Assay

Linear scratches were created in the single layers of confluent FaDu cells, kept in 6-well microplates, using 200-*μ*L disinfected pipette tips. PBS (Phosphate Buffer Saline) was used to clear the cellular debris, and microscopic monitoring of the wound closure was accomplished separately at 0 h and 24 h following injury.

### 2.13. Immunofluorescence (IF) Assay

In a 24-well microplate, FaDu cells were inoculated on coverslips, fixed in paraformaldehyde (4%) for 15 min, and, where necessary, permeabilized for 20 min using Triton X-100 (0.5%). The coverslips were incubated overnight in 1 : 100 anti-E-cadherin (#3195), 1 : 100 anti-N-cadherin (#13116; both Cell Signaling Technology, Danvers, USA) together with 1 : 500 anti-Vimentin (ab92547, Abcam, Cambridge, UK) antibodies at 4°C after a 1 h blockage in goat serum (Beyotime, Shanghai, China). On the following day, these coverslips were processed for 1 h using a 1 : 250 fluorescent secondary antibody (ab150078, Abcam, Cambridge, UK) and subsequent 5 min processing with DAPI (Beyotime, Shanghai, China) at ambient temperature. After applying the antifade mounting medium to slow the fluorescent quenching, a laser scanning confocal microscope was utilized to surveil and photograph the coverslips.

### 2.14. Animal Experiments

Murine models of popliteal LN metastasis were created using nude male BALB/c mice aged 5 weeks (Tengxin, Chongqing, China), reared in the Animal Laboratory of Chongqing Medical University under SPF conditions. The mice were categorized randomly into two groups (5 mice per group) and exposed to injection in the footpads with 2 × 10^6^ FaDu cells transfected with empty vector (FaDu-Control cells) or FaDu cells with stable overexpression of RBM24 (FaDu-OE-RBM24 cells) suspended in 30 *μ*L PBS. A caliper was utilized to quantify the tumor dimensions, and the murine body weight was evaluated at 5 d intervals. Fifteen days following cellular implantation, an Indigo 2.0.5.0 (Berthold Technologies, Germany) was utilized to assess the tumor evolution and LN metastasis through in-vivo imaging. The mice were killed twenty-five days later, and their primary tumor tissues, along with LNs, were harvested for embedding in paraffin. The computational formula for tumor volume was 0.5 × length × width^2^. For IHC tissues staining, 1 : 200 anti-RBM24 (ab94567), 1 : 250 anti-Vimentin (ab92547; both Abcam, Cambridge, UK), 1 : 100 anti-Twist1 (AF4009, Affinity), and 1 : 400 anti-E-cadherin (#3195, Cell Signaling Technology, MA, USA) antibodies were adopted. H&E (hematoxylin and eosin) staining was used for LNs. Moreover, the murine experiments' approval was acquired from the First Affiliated Hospital of Chongqing Medical University's Ethics Committee.

### 2.15. Statistical Analysis

This study utilized SPSS 22.0 (IBM Corp, Armonk, NY, USA) and GraphPad Prism 8.0 (GraphPad, San Diego, USA) to perform statistical analysis. Student's *t*-test or the Mann–Whitney *U* test was used to assess the significance of differences between two groups, while one-way ANOVA was used to compare three or more groups. Correlations between the IHC expression of RBM24 and clinicopathological characteristics were assessed using Pearson's chi-squared test or Fisher's exact test. The Kaplan-Meier analysis with the logrank test was adopted to evaluate overall survival (OS). The data are represented as the mean ± standard error of the mean (SEM) values, and a *p*value < 0.05 was regarded as statistically significant.

## 3. Results

### 3.1. RBM24 Was Downregulated in HSCC Patients with LN Metastasis

**Figure 1:**
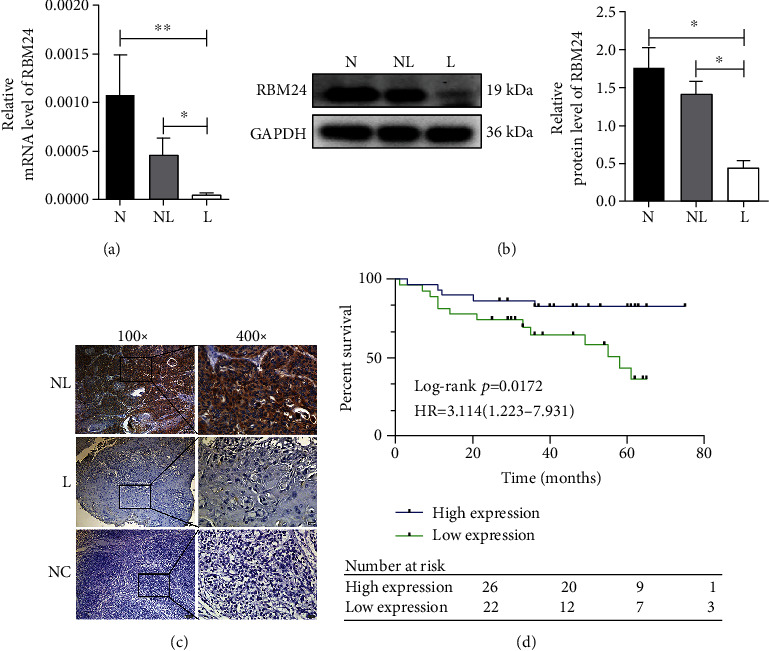
Downregulated RBM24 among the HSCC patients presenting LN metastasis was linked to poor outcomes. (a and b) The qRT-PCR and WB outcomes for RBM24 level in HSCC. (c) Typical IHC outcomes for RBM24; staining of HSCC tissue specimens from the LN metastatic and nonmetastatic populations. PBS served as the negative control to substitute for the primary antibody, where the magnification was 100× for the left panel and 400× for the right panel. (d) Kaplan-Meier overall survival plots for HSCC patients (*n* = 57) expressing RBM24 highly and lowly. ^∗^*p* < 0.05, ^∗∗^*p* < 0.01. Abbreviations: N stands for normal tissues; NL represents the tumor tissues from the non-LN metastatic population; L represents the tumor tissues from LN metastatic population; NC stands for negative control.

In this study, transcriptome sequencing was performed to identify the differentially expressed genes (DEmRNAs) in HSCC patients who had or did not have LN metastasis in our former research [23, 24]. RBM24 was identified as one downregulated gene in the LN metastasis group. Several members of RRM family have been reported to play vital role in cancer metastasis [25, 26], and a bioinformatics study based on data from TCGA database suggested RBM24 might be a prognostic-related gene in head and neck squamous cell carcinoma [27]. But, the role of RBM24 in HSCC has not been expounded yet. Subsequently, this study detected the RBM24 expression at the mRNA and protein levels. Our study found that the mRNA level of RBM24 in eight LN metastasis cases was lower than in both the eight adjacent normal cases (*p* < 0.01) and the 8 cases that did not have LN metastasis (*p* < 0.05) (Figure 1(a)). Consequently, the RBM24 protein level was lower in five LN metastasis cases than in the other two groups (five cases without LN metastasis and five corresponding adjacent normal tissues) through WB analysis (*p* < 0.05) (Figure 1(b)). In addition, RBM24 expression in tissues was examined by IHC analysis.  Figure 1(c) showed the differential staining of RBM24 in HSCC tissues and the staining of RBM24 in non-LN metastatic tissues was stronger than those in LN metastatic tissues. The proportion of tissues with low RBM24 expression was higher in those undergoing LN metastasis (25/41 [61.0%]) than those without LN metastasis (2/16 [12.5%]) (*p* < 0.001; Table 2). Moreover, this study evaluated the associations between RBM24 expression and clinical characteristics in 57 HSCC patients and found that RBM24 expression was correlated with tumor stage (*p* < 0.01) as well as LN stage (*p* < 0.001) but not with age, sex, or pathological stage (Table 3). According to Kaplan-Meier survival analysis, those who had low RBM24 expression had poor overall survival (*p* < 0.05) (Figure 1(d)). All the findings suggested that RBM24 was downregulated in HSCC patients with LN metastasis, showing a close association with poor prognosis.

### 3.2. Inhibition of RBM24 Stimulated the Proliferation, Migration, and Invasion of FaDu Cells

**Figure 2:**
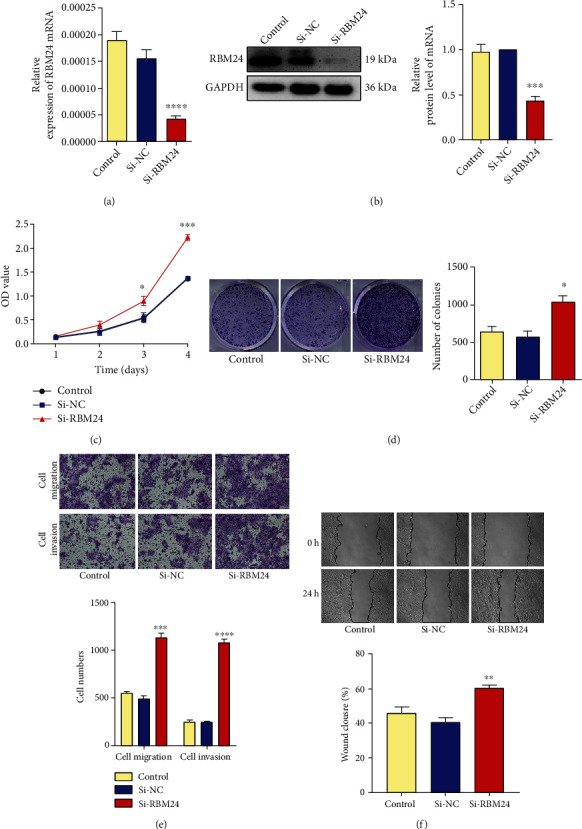
Facilitation of the FaDu cell multiplication, motility, and invasiveness by the RBM24 knockdown. (a and b) Lowered protein and mRNA expressions of RBM24 in the FaDu cells silenced for RBM24. (c) CCK-8 assay outcomes for the FaDu cell vitality at four varying time points. (d) Colony formation assay outcomes for the multiplicative potential of FaDu cells. (e) Transwell assay outcomes for the invasive and migratory potential of FaDu cells under a magnification of 100×. (f) Wound healing assay outcomes for the FaDu cell motility under a magnification of 100×. ^∗^*p* < 0.05, ^∗∗^*p* < 0.01, ^∗∗∗^*p* < 0.001, ^∗∗∗∗^*p* < 0.0001. The data are represented as the mean ± SEM values from no less than three independently experiments.

qRT-PCR combined with WB analysis was conducted to validate the expression of RBM24 to investigate RBM24 biofunctionality in FaDu cells. The RBM24 siRNA#1, #2, and #3, together with the Si-NC (control siRNA), were used to transfect the FaDu cells. The knockdown of RBM24 was found to be most efficient with siRNA#3 (Si-RBM24) (Supplementary Figures 1(a) and 1(b), Figures 2(a) and 2(b)) and chosen for further investigations. According to the colony formation and proliferation assay, the FaDu cell multiplication was facilitated by suppressing RBM24 (Figures 2(c) and 2(d)). Next, more FaDu cells went through the upper chambers in the Si-RBM24 group compared with the Control and Si-NC group according to transwell migration and invasion assays (Figure 2(e)), indicating the RBM24 knockdown improved the invasiveness and motility of FaDu cells. Besides, the wound healing assay showed knockdown of RBM24 increased the migration speed of FaDu cells at 24 hours after injury (Figure 2(f)), suggesting the RBM24 knockdown enhanced wound healing capacity of FaDu cells. In conclusion, deletion of RBM24 could promote the proliferation, invasion, and migration of FaDu cells.

### 3.3. Overexpression of RBM24 Restrained the Proliferation, Migration as well as Invasion of FaDu Cells

**Figure 3:**
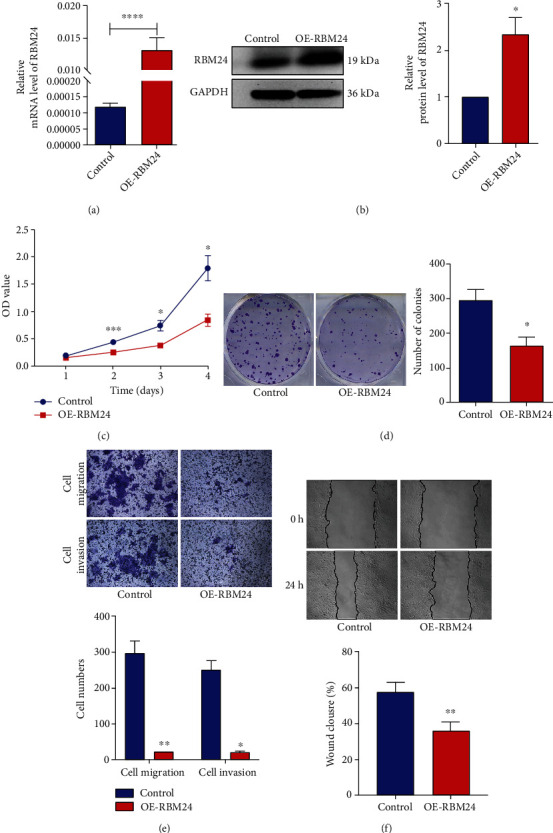
Suppression of the FaDu cell multiplication, motility, and invasiveness by the RBM24 overexpression. (a and b) Elevated protein and mRNA expressions of RBM24 in the FaDu-OE-RBM24 cells. (c) CCK-8 assay outcomes for the FaDu cell vitality at four varying time points. (d) Colony formation assay outcomes for the multiplicative potential of FaDu cells. (e) Transwell assay outcomes for the FaDu cell invasive and migratory potential under 100× magnification. (f) Wound healing assay outcomes for the FaDu cell motility under 100× magnification. ^∗^*p* < 0.05, ^∗∗^*p* < 0.01, ^∗∗∗∗^*p* < 0.0001. The data are represented as the mean ± SEM values from no less than three independently experiments.

This study generated FaDu-OE-RBM24 cells by infection with lentiviral particles and evaluated the RBM24 overexpression efficiency using qRT-PCR (Figure 3(a)) and WB (Figure 3(b)) analyses. As the colony formation and proliferation assays indicated, the RBM24 overexpression in FaDu cells led to multiplication reduction (Figures 3(c) and 3(d). The transwell assay exhibited less FaDu cells went through the upper chambers in the OE-RBM24 group compared with the Control group (Figure 3(e)), indicating RBM24 overexpression led to invasive and migratory inhibition of FaDu cells. Besides, wound healing assay showed the RBM24 overexpression decreased the migration speed of FaDu cells at 24 hours after injury (Figure 3(f)), suggesting overexpression of RBM24 led to migratory inhibition of FaDu cells. These findings suggested the regulatory role of RBM24 in the FaDu cell multiplicative, invasive, and migratory potential.

### 3.4. Overexpression of RBM24 Downregulated Twist1 and Hindered EMT in FaDu Cells

**Figure 4:**
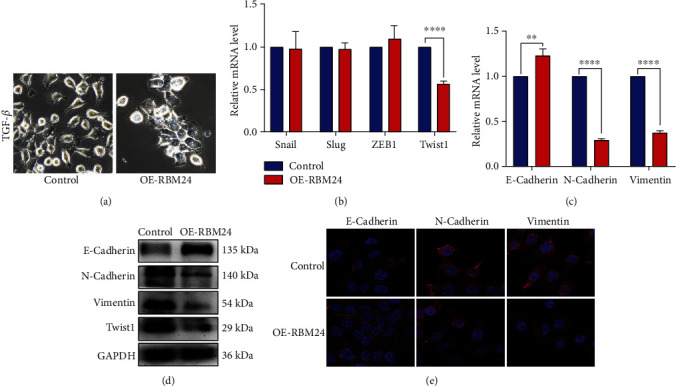
Twist1 downregulation and EMT suppression in FaDu cells led by the RBM24 overexpression. (a) Representative images of cell morphology after 48 h treatment using TGF*β* (200× magnification). (b) The qRT-PCR outcomes for Slug, Snail, ZEB1, and Twist1 mRNA expressions in the FaDu-OE-RBM24 and FaDu-Control cells. (c) The qRT-PCR outcomes for N-cadherin, E-cadherin, and Vimentin mRNA expressions in the FaDu-OE-RBM24 and FaDu-Control cells. (d) The WB outcomes for the N-cadherin, E-cadherin, Vimentin, and Twist1 protein expressions. (e) The confocal immunofluorescent microscopy-based observations of N-cadherin, E-cadherin, and Vimentin levels under a magnification of 400×. ^∗∗^*p* < 0.01, ^∗∗∗∗^*p* < 0.0001. The data are represented as the mean ± SEM values from no less than three independently experiments.

The pivotal effects of EMT on head and neck squamous cell carcinoma metastasis have been established universally [28], and extensive studies have reported the role of TGF*β* as a potent elicitor of EMT [29]. In our work, loss of RBM24 was found to elicit the malignant HSCC progression. Hence, the linkage of RBM24 to the TGF*β*-triggered EMT was investigated. After eliciting EMT in FaDu cells through a 48 h treatment using TGF*β* (20 ng/mL), more FaDu-Control cells showed spindle shape and loss of intercellular adhesion (Figure 4(a)), indicating FaDu-Control cells underwent EMT. Then the mRNA levels of EMT-TFs (EMT-triggering transcriptional factors) were assessed to clarify the responders to the overexpression of RBM24. As displayed in Figure 4(b), Twist1 was RBM24's downstream effector, while changes in the Slug, Snail, and ZEB1 expressions were inapparent. RBM24 overexpression contributed to a decline in Twist1 protein expression (Figure 4(d)). Correspondingly, the mRNA and protein expressions of E-cadherin were elevated in FaDu-OE-RBM24 cells, whereas N-cadherin and Vimentin declined (Figures 4(c) and 4(d), implying TGF*β*-triggered EMT suppression by the RBM24 overexpression. A further confocal immunofluorescent assay showed the fluorescent signal of E-cadherin was stronger while the fluorescent signals of N-cadherin and Vimentin were weaker in the FaDu-OE-RBM24 cells compared with the FaDu-Control cells (Figure 4(e)), which revealed that the RBM24 overexpression increased E-cadherin and decreased N-cadherin and Vimentin. As indicated by the preceding findings, RBM24 was capable of suppressing the TGF*β*-triggered EMT event and lowered the Twist1 level.

### 3.5. Overexpression of Twist1 Could Reverse the Inhibitory Impacts of RBM24 on Invasion, Migration, and EMT in FaDu Cells

**Figure 5:**
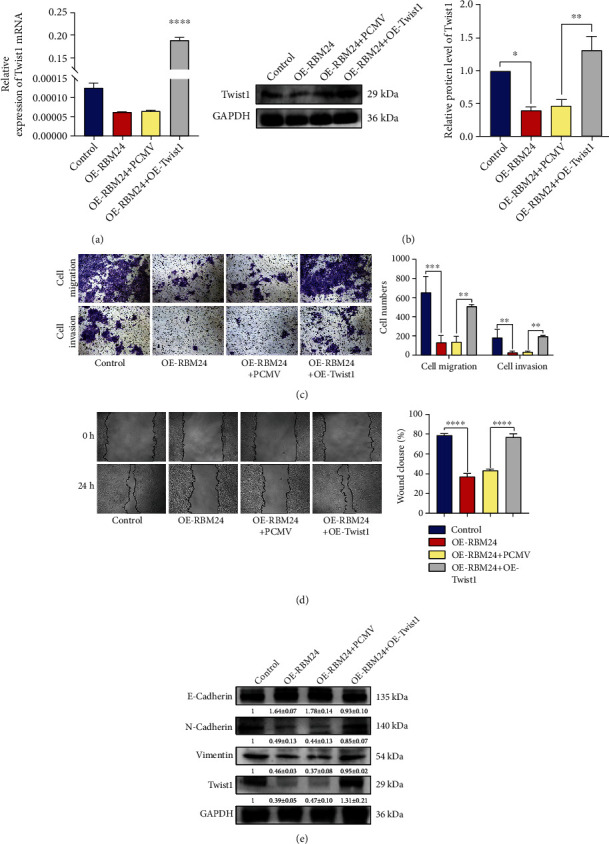
Reversal of RBM24's suppressive actions against the FaDu cell migration, invasiveness, and EMT by Twist1 overexpression. (a and b) Protein and mRNA expressions of Twist1 in the investigated cells. (c) Transwell assay outcomes for the FaDu cell invasive and migratory potential under 100× magnification. (d) Wound healing assay outcomes for the FaDu cell motility under 100× magnification. (e) N-cadherin, E-cadherin, Vimentin, and Twist1 protein levels in the investigated cells. ^∗^*p* < 0.05, ^∗∗^*p* < 0.01, ^∗∗∗^*p* < 0.001, ^∗∗∗∗^*p* < 0.0001. The data are represented as the mean ± SEM values from no less than three independently experiments.

Given the essential function of Twist1 in the HSCC evolution, our study explored whether it is indispensable for RBM24's actions in HSCC cells by transfecting a Twist1-expressing plasmid and a control plasmid (PCMV) into the FaDu-OE-RBM24 cells. The expression of Twist1 was validated by qRT-PCR combined with WB analysis (Figures 5(a) and 5(b). Transwell and wound healing assays showed the cellular invasiveness, motility were repressed, and WB analysis showed the protein level of E-cadherin was elevated whereas the levels of N-cadherin and Vimentin declined by RBM24 overexpression. However, the Twist1 overexpression reversed these effects (Figures 5(c)–5(e)). Thus, the RBM24 overexpression was capable of inhibiting the cellular migratory/invasive potential and EMT process through adverse Twist1 modulation.

### 3.6. Overexpression of RBM24 Hindered HSCC Tumor Growth as well as LN Metastasis In-Vivo

**Figure 6:**
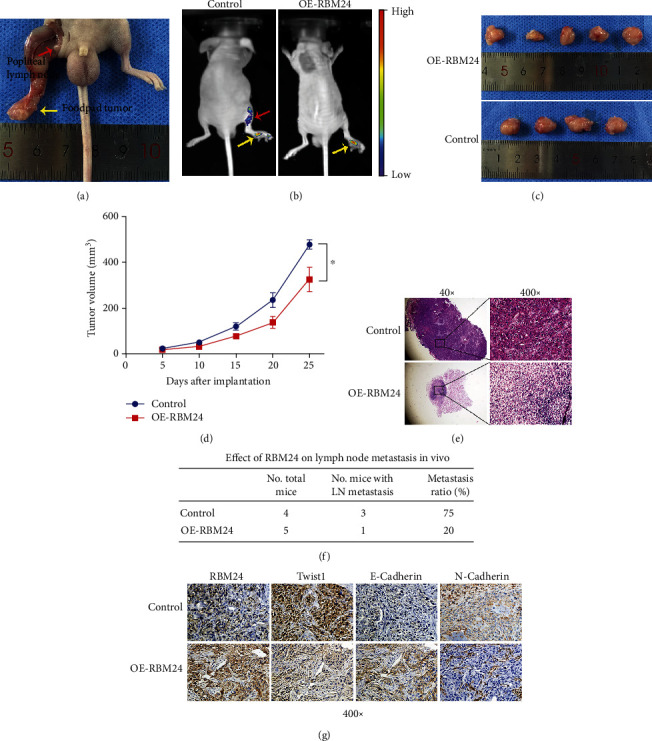
In-vivo inhibition of the HSCC evolution and metastasis to LNs by the RBM24 overexpression. (a) Typical model illustrating the metastasis to popliteal LNs. (b) Typical fluorescence micrographs of FaDu-OE-RBM24 and FaDu-Control cell-inoculated mice. The footpad tumors are represented by yellow arrows, whereas red arrows represent the metastases to popliteal LNs. (c) Excised tumor images of the FaDu-OE-RBM24 cell-inoculated mice (*n* = 5) and the corresponding FaDu-Control mice (*n* = 4). (d) Tumor growth graphs of the FaDu-OE-RBM24 group (*n* = 5) and the FaDu-Control group (*n* = 4). (e) Typical H&E-stained micrographs of metastatic LNs for the FaDu-OE-RBM24 mice (*n* = 5) and the FaDu-Control mice (*n* = 4), where the magnification was 40× for the left panel and 400× for the right panel. (f) Percentages of LN metastatic mice in the FaDu-OE-RBM24 group (*n* = 5) versus the FaDu-Control group (*n* = 4). (g) Typical micrographs of IHC-stained tumor cells in the FaDu-OE-RBM24 and FaDu-Control groups. ^∗^*p* < 0.05.

The nude male BALB/c mice were inoculated with FaDu-OE-RBM24 or FaDu-Control cells at the footpads to investigate RBM24 level impacts on the HSCC evolution and metastasis to LNs in-vivo (Figure 6(a)). Both groups exhibited distinct fluorescent signals in the murine footpads (Figure 6(b)). Nevertheless, these signals were noted in the popliteal fossa, indicating metastasis to LNs in the FaDu-Control group. Slower tumor growth was detected in the FaDu-OE-RBM24 mice compared to the FaDu-Control mice (Figure 6(d)). Besides, the FaDu-OE-RBM24 group also exhibited lower tumor volumes (Figure 6(c)). The metastatic cell counts in LNs were reduced by the stable RBM24 overexpression (Figure 6(e)). According to Figure 6(f), metastasis to LNs was noted in one-fifth of the FaDu-OE-RBM24 mice, while it was absent in three-fourths of the FaDu-Control mice. IHC assay found higher RBM24 and E-cadherin levels yet lower Twist1 and Vimentin levels in the FaDu-OE-RBM24 group's tumors compared to the FaDu-Control tumors (Figure 6(g)). Thus, based on the in-vitro surveillance, it was presumed that the RBM24 overexpression downregulated Twist1 in-vivo.

## 4. Discussion

RBPs exert crucial functions in regulating pathways involved in numerous aspects of RNA metabolism, including transcription, alternative splicing, modification, stability, subcellular localization, translation, and decay [11, 30]. The implication of abnormal RBPs expression (critical gene regulatory proteins) or their mutations and binding sites in RNA targets has been reported in diverse human diseases containing carcinomas [31]. According to the latest review, RBPs exert a role in carcinoma metastasis through mechanotransduction modulation [32].

RBM24, an RRM family member, is a p53 pathway participant through stability modulation of p21 mRNA and interplay with p53 in diverse human carcinoma cells [13, 33]. The modulator role of RBM24 in the p63 expression is achieved by intrinsic mRNA stability [34]. The antitumor role of RBM24 in a few carcinomas is noteworthy [16–18]. Despite these, there is insufficient knowledge about its effects on carcinogenesis and evolution, particularly in hypopharyngeal carcinomas. In our preliminary work, the genes showing altered expression were investigated through RNA sequencing among HSCC patients with or without metastasis to LNs, and RBM24 was one of the downregulated genes. The downregulation of RBM24 was confirmed among HSCC patients showing metastasis to LNs. Besides, lower expression of RBM24 was related to several clinicopathological characteristics and worse overall survival, indicating that RBM24 could be both a biomarker for early diagnosis and a potential target for treating HSCC. For the in-vitro biofunctionality evaluation of RBM24, its level was adjusted in FaDu cells with lentiviral particles and siRNAs. The results verified that RBM24 could suppress the cellular multiplicative, migratory, and invasive potential and the EMT event in-vitro. Most importantly, the probable function of RBM24 was as a tumor inhibitor against the HSCC evolution.

Upon initial diagnosis, metastasis to cervical LNs was usually detected in the HSCC patients, resulting in therapeutic failure and dissatisfied overall survival [3, 35–37]. Thus, it is imperative to explore the mechanisms concerning HSCC evolution and LN metastasis. The pivotal effects of EMT on carcinoma progression have been established universally. The features of EMT include the polarity depletion of epithelial cells and their phenotypic transformation into mesenchymal [38]. Because of the previous processes, cancer cells can escape from the original site to metastasize distantly. EMT is defined by lowered E-cadherin (epithelial marker) and elevated Vimentin and N-cadherin (mesenchymal markers) levels [39]. EMT has recently been linked tightly to LN metastasis [40, 41]. EMT-TFs are the chief EMT regulators, Twist1 being a kernel factor [29]. According to the latest research, Twist1 is an oncogene whose aberrant overexpression promotes carcinogenesis, evolution, and metastasis. Twist1 can facilitate carcinoma evolution through the cancer cell multiplication enhancement and apoptosis inhibition, leading to strengthened chemotherapy susceptibility of cancer cells and an extended colony of cancer stem cells to prompt the metastasis and invasiveness [42, 43]. Our study investigated the links between RBM24 expression and TGF*β*-induced EMT, given the significance of EMT in tumor metastasis and former research showing that RBM24 overexpression could upregulate E-cadherin expression but downregulate Vimentin expression in HCT116 human CRC cells [17]. The present study validated on HSCC FaDu cells that the expressions of a few representative EMT markers, along with Twist1, could be modulated by RBM24. Thus, RBM24 may target Twist1 to suppress the TGF*β*-triggered EMT and HSCC metastasis to LNs. Rescue experiments were conducted where Twist1 was overexpressed in FaDu cells to validate this assumption. Reversal of cellular invasiveness, migration, and EMT was found after Twist1 overexpression. Besides, RBM24 overexpression inhibited HSCC evolution and metastasis to LNs in-vivo.

Further IHC staining revealed the corresponding alterations in a few EMT markers and Twist1 levels. Thus, RBM24 overexpression probably suppresses the HSCC evolution and diminishes the LN metastasis through Twist1 level repression. However, further explorations are required to validate the Twist1-binding ability of RBM24 and clarify the precise mechanism. Deeper probing is required to specify the mechanisms of RBM24 and Twist1 interaction to modulate the LN metastasis and the EMT.

## 5. Conclusion

To conclude, RBM24, as a novel tumor inhibitor gene, inhibits the LN metastasis and EMT by the downregulation of the Twist1 in HSCC. Hence, the RBM24 and Twist1 probably serve as diagnostic biomarkers and therapeutic targets for HSCC patients presenting LN metastasis.

## Figures and Tables

**Table 1:** Primer sequences.

Gene name	Primer sequences (5′-3′)
RBM24	Forward: GAA CCT GGC ATA CTT AGG AGC A Reverse: AGG TCT TTG TAT AAG GGC TGG A
E-cadherin	Forward: TGC CCA GAA AAT GAA AAA GG Reverse: GTG TAT GTG GCA ATG CGT TC
N-cadherin	Forward: GAC AAT GCC CCT CAA GTG TT Reverse: CCA TTA AGC CGA GTG ATG GT
Vimentin	Forward: GAG AAC TTT GCC GTT GAA GC Reverse: GCT TCC TGT AGG TGG CAA TC
Twist1	Forward: ACA AGC TGA GCA AGA TTC AGA CC Reverse: TCC AGA CCG AGA AGG CGT AG
Snail	Forward: GCG AGC TGC AGG ACT CTA AT Reverse: CCT CAT CTG ACA GGG AGG TC
Slug	Forward: TGA TGA AGA GGA AAG ACT ACAG Reverse: GCTCACATATTCCTTGTCACAG
ZEB1	Forward: TGC ACT GAG TGT GGA AAA GC Reverse: TGG TGA TGC TGA AAG AGA CG
GAPDH	Forward: GGA GTC CAC TGG CGT CTT CA Reverse: GTC ATG AGT CCT TCC ACG ATA CC

**Table 2:** The immunohistochemical expression of RBM24 in HSCC patients with lymphatic metastasis and without metastasis.

Tissue	Number of patients	Expression of RBM24		*P* value
		High	Low	
Lymphatic metastasis	41	16 (39.0%)	25 (61.0%)	<0.001
Non-lymphatic metastasis	16	14 (87.5%)	2 (12.5%)	

*P* values are from *χ*^2^ test or Fisher's exact test and were statistically significant when < 0.05.

**Table 3:** Relationship between RBM24 expression and clinicopathological characteristics of 57 HSCC patients.

Category	Number of patients	Expression of RBM24		*P* value
		High	Low	
Age (years old)				0.63
(i) <60	23	13	10	
(ii) ≥60	34	17	17	
Gender				1
(i) Male	56	29	27	
(ii) Female	1	1	0	
T stage				<0.01
(i) T1 + T2	11	10	1	
(ii) T3 + T4	46	20	26	
N stage				<0.001
(i) N0 + N1	24	20	4	
(ii) N2 + N3	33	10	23	
Pathological stage				0.82
(i) High differentiation	14	7	7	
(ii)Moderate or poor differentiation	43	23	20	

*P* values are from *χ*^2^ test or Fisher's exact test and were statistically significant when < 0.05. Abbreviations: T stage, tumor stage; N stage, lymph node stage.

## Data Availability

The corresponding author, on reasonable request, offered the obtained data.

## Supplementary Materials

Supplementary Materials Supplementary Figure 1 SiRNA#3 (Si-RBM24) exhibited the highest RBM24 knockdown efficiency. (a) mRNA level of RBM24 in FaDu cells transfected with RBM24 siRNAs. (b) Protein expression level of RBM24 in FaDu cells transfected with RBM24 siRNAs. ^∗∗^*p* < 0.01.
