# Supplementary material for: RBM24 Mediates Lymph Node Metastasis and Epithelial-Mesenchymal Transition in Human Hypopharyngeal Squamous Cell Carcinoma by Regulating Twist1
Source: J Oncol. 2022 Sep 29;2022:1205353. doi: 10.1155/2022/1205353 (PMC9536977; doi:10.1155/2022/1205353)
Supplement: Supplementary Materials — Supplementary Figure 1 SiRNA#3 (Si-RBM24) exhibited the highest RBM24 knockdown efficiency. (a) mRNA level of RBM24 in FaDu cells transfected with RBM24 siRNAs. (b) Protein expression level of RBM24 in FaDu cells transfected with RBM24 siRNAs. ∗∗p < 0.01. [file 1205353.f1.docx]

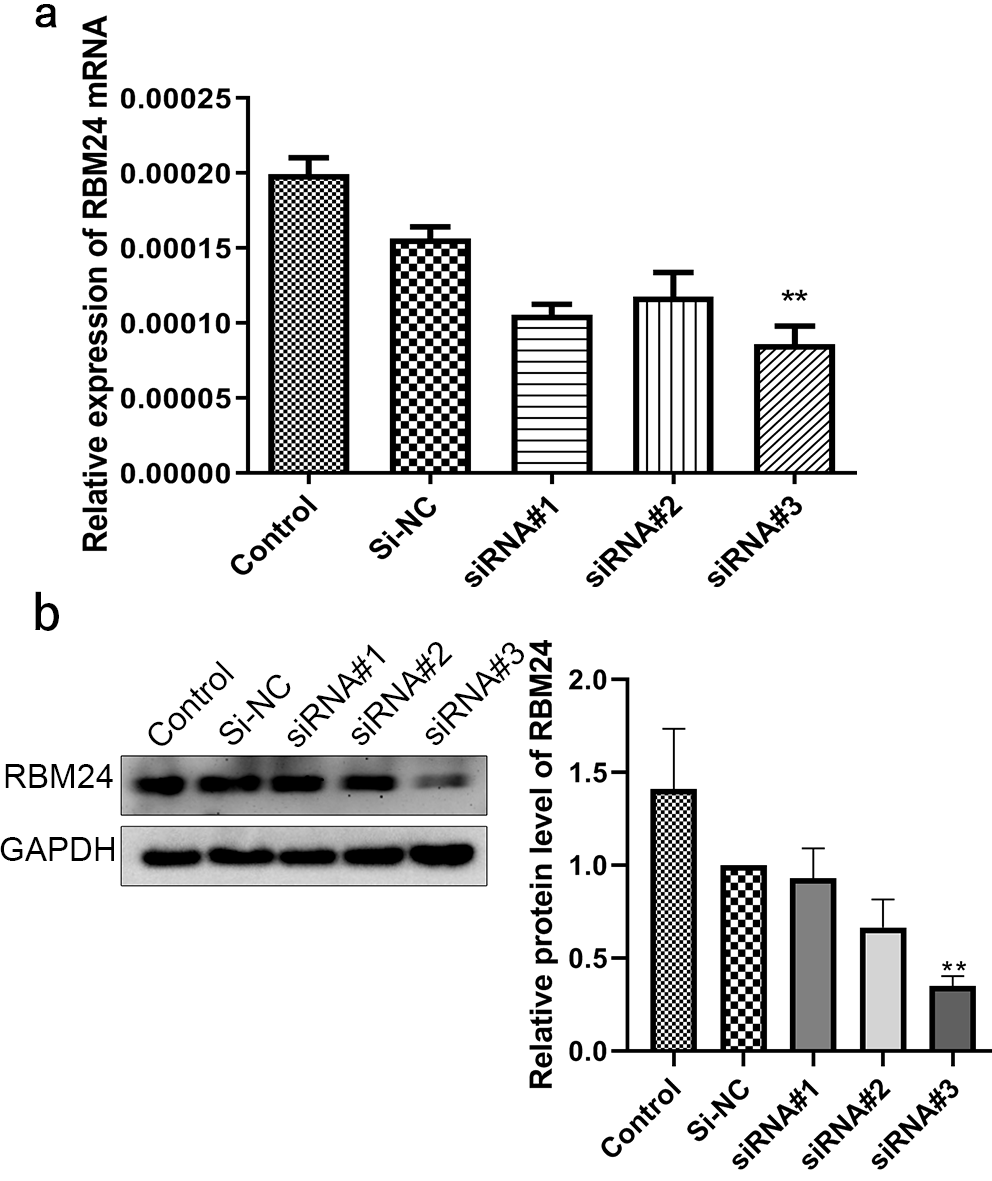


**Supplementary Fig. 1** SiRNA#3 (Si-RBM24) exhibited the highest RBM24 knockdown efficiency. **(a)** mRNA level of RBM24 in FaDu cells transfected with RBM24 siRNAs. **(b)** Protein expression level of RBM24 in FaDu cells transfected with RBM24 siRNAs. **p < 0.01.
